# 3D-Planned Posterior Open-Wedge Glenoid Osteotomy Using a Patient-Specific Cutting Guide in Posterior Shoulder Instability: A Technical Note

**DOI:** 10.7759/cureus.97902

**Published:** 2025-11-26

**Authors:** Bernard De Geofroy, Paul Teixeira, Romain Pacull, Thibaut Poujade, Camille Choufani

**Affiliations:** 1 Orthopaedics and Traumatology, Laveran Military Hospital, Marseille, FRA; 2 Orthopaedics and Traumatology, Sainte-Anne Military Hospital, Toulon, FRA; 3 Orthopaedics, Skylab, Haute-Goulaine, FRA

**Keywords:** glenoid osteotomy, patient-specific instrumentation, posterior shoulder instability, shoulder biomechanics, three-dimensional planning

## Abstract

Posterior shoulder instability remains a rare but disabling condition often underdiagnosed due to subtle symptoms and delayed presentation. Excessive glenoid retroversion is a key anatomical determinant of posterior humeral head subluxation. When isolated soft tissue repair fails, posterior opening glenoid osteotomy can restore physiological glenoid version. Traditional freehand or fluoroscopic techniques, however, are technically demanding and prone to variability. This technical note describes a 3D-planned posterior open-wedge glenoid osteotomy using a patient-specific cutting guide (PSI) to correct excessive retroversion in posterior instability. Preoperative 3D computed tomography (CT) planning (ONE®, Newclip Technics, Haute-Goulaine, France) allows the virtual correction of retroversion and design of a custom guide conforming to the posterior glenoid surface. The PSI ensures accurate orientation, safe osteotomy execution, and faithful translation of the preoperative plan. Fixation is achieved with a low-profile locking plate, enabling early mobilization.

## Introduction

Posterior shoulder instability remains a rare but disabling condition, often underrecognized due to subtle symptoms and frequent diagnostic delay. Among the anatomical factors involved, excessive glenoid retroversion or glenoid dysplasia has been identified as a major determinant of humeral head decentering [1,2]. When isolated capsulolabral repair proves insufficient, posterior glenoid osteotomy represents an anatomical surgical option aimed at restoring physiological version [3]. Since the initial description by Scott [4], several case series have reported variable outcomes. Graichen et al. demonstrated satisfactory mid-term results in atraumatic instabilities, albeit with the development of glenohumeral osteoarthritis in one quarter of cases [5]. More recently, Waltenspül et al. reported, after more than 15 years of follow-up, a high rate of recurrence and nearly universal arthritic progression, suggesting that correction of retroversion alone is insufficient to restore normal glenohumeral biomechanics [6]. These limitations have prompted a more comprehensive, three-dimensional understanding of posterior instability. Scapular dysmorphia (defined by the combination of glenoid retroversion and a high, horizontally oriented acromion) has emerged as a key factor promoting static posterior subluxation [7-9]. In this context, Gerber et al. recently demonstrated the feasibility of global scapular reshaping using combined acromial and glenoid osteotomies planned in 3D, allowing durable recentering of the humeral head [10]. The recent development of advanced 3D planning tools and patient-specific cutting guides has further improved the precision and reproducibility of these technically demanding procedures. This article describes a 3D-planned posterior opening glenoid osteotomy using a patient-specific cutting guide (PSI), with the objective of restoring physiological glenoid version while ensuring safe, accurate, and reproducible execution of the procedure in posterior instabilities associated with excessive glenoid retroversion.

## Technical report

Concept and materials

The procedure aims to correct pathological glenoid retroversion through a posterior opening wedge osteotomy guided by a patient-specific cutting guide. This 3D-printed guide, designed from preoperative computed tomography (CT) data, ensures the precise orientation and reproducible execution of the planned correction. Guides are manufactured using biocompatible, sterilizable polymers (polyamide or resin) shaped to the posterior scapular contour. The osteotomy gap is stabilized by a low-profile plate until consolidation.

Preoperative planning and guide design

**Figure 1:**
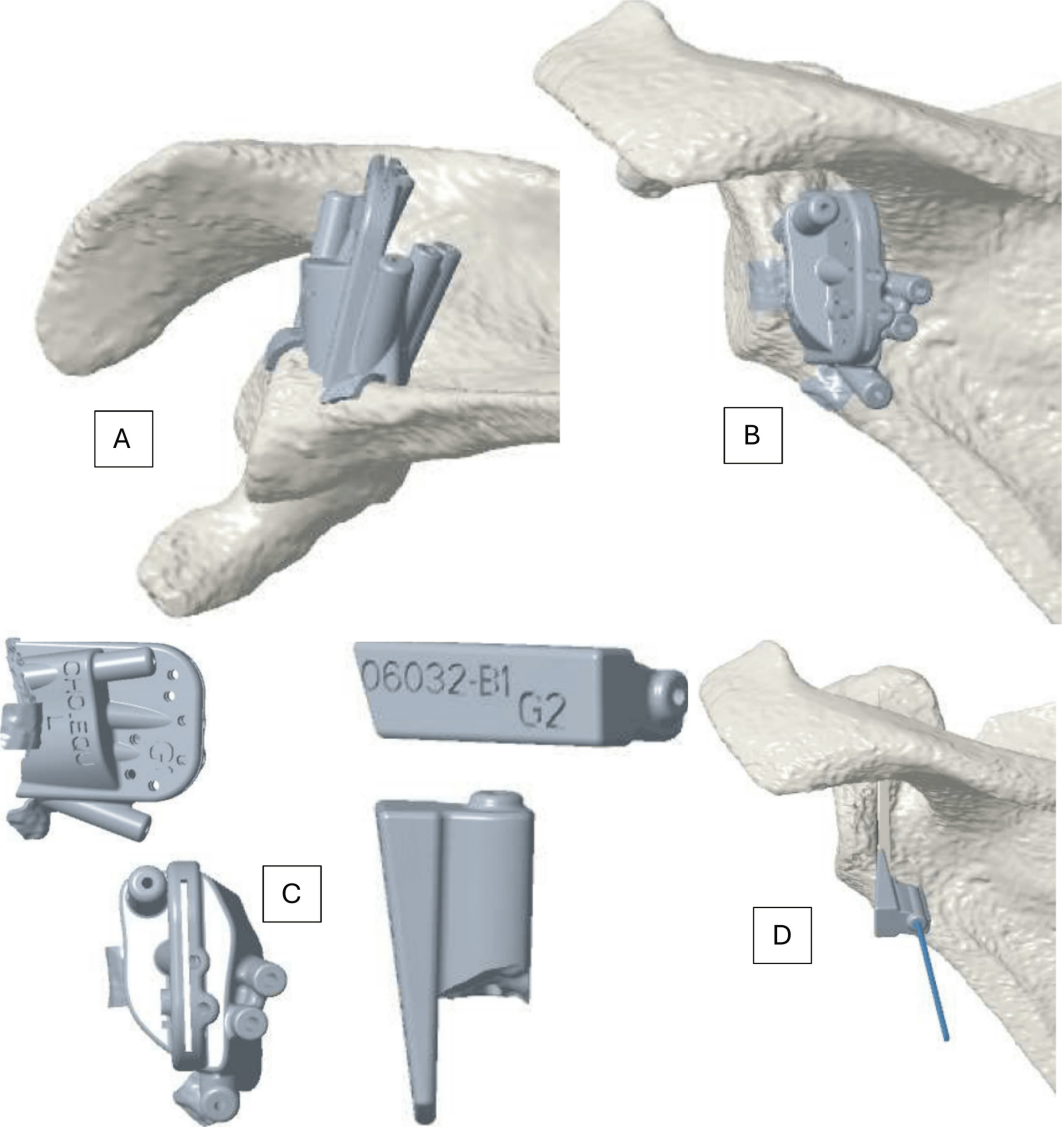
3D planning with patient-specific cutting guide positioning on the posterior aspect of the glenoid (A) Inferior view of the scapula. (B and D) Posterior view of the scapula. (C) Patient-specific cutting guide and wedge spreader.

Preoperative planning was based on a high-resolution CT scan of the affected shoulder, acquired with a slice thickness ≤1 mm and encompassing the entire scapula. The Digital Imaging and Communications in Medicine (DICOM) data were imported into a dedicated 3D planning software (ONE®, Newclip Technics, Haute-Goulaine, France) to reconstruct a three-dimensional model of the scapula and glenoid. The native glenoid version and inclination were measured according to Friedman's line. As this report is based on a single patient, illustrative case data detailing the 3D planning and corrective osteotomy are provided (Figure 1).

**Figure 2:**
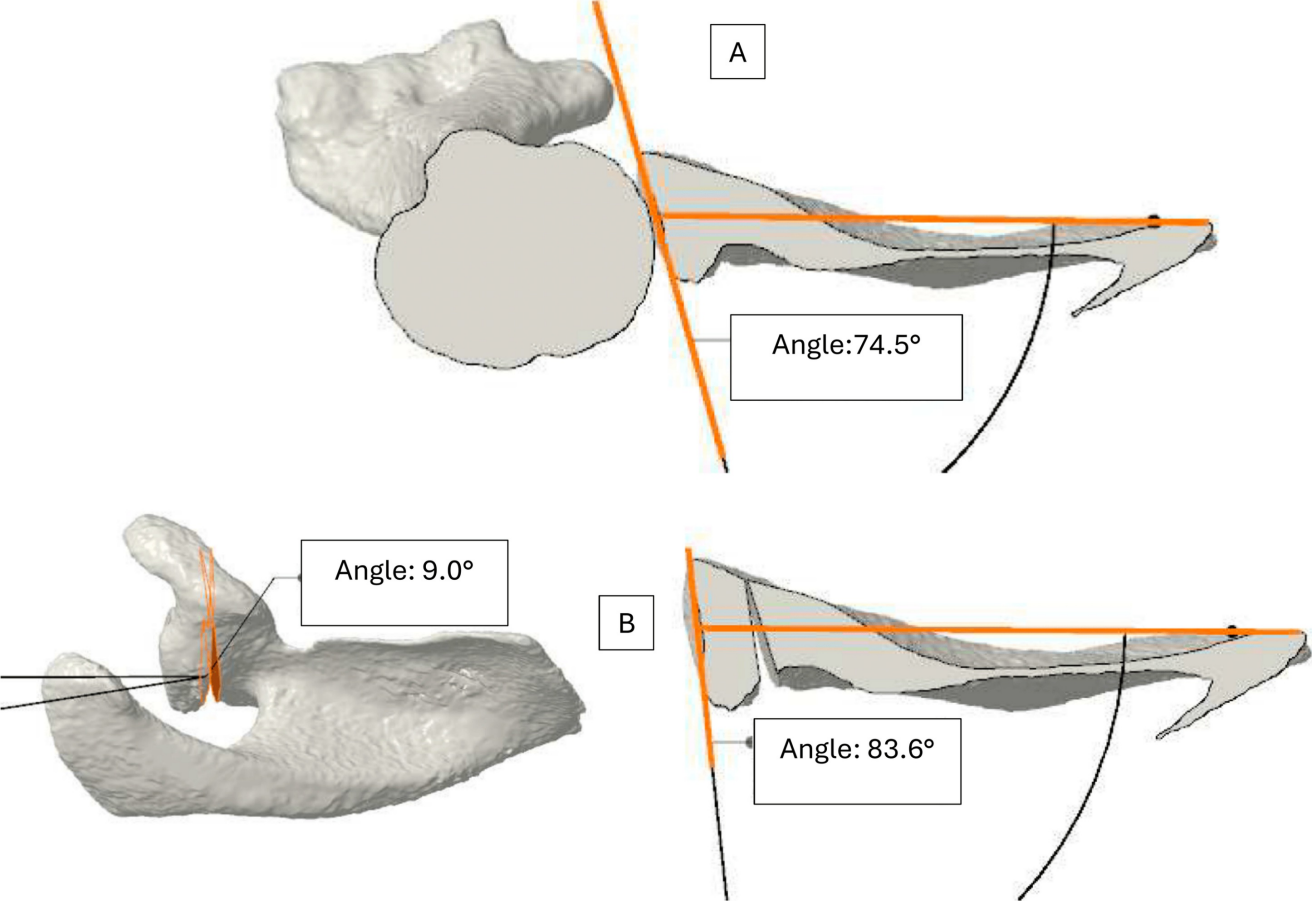
Preoperative planning showing correction from an angle of 15.6° to 6.4° (opening of 9° and 3.7 mm) (A) Preoperative glenoid orientation. (B) Postoperative glenoid orientation.

The acromial values (sagittal inclination: 62°; normalized posterior acromial height: 0.6; posterior coverage: 66.3°) were measured to determine whether an acromial correction should be associated with the glenoid osteotomy [11]. A patient-specific corrective osteotomy was virtually simulated to restore a physiological glenoid version, typically between 0° and 5° of retroversion, depending on the contralateral side. The magnitude and location of the posterior opening wedge were determined to achieve this target orientation while preserving maximal subchondral bone stock (Figure 2).

**Figure 3:**
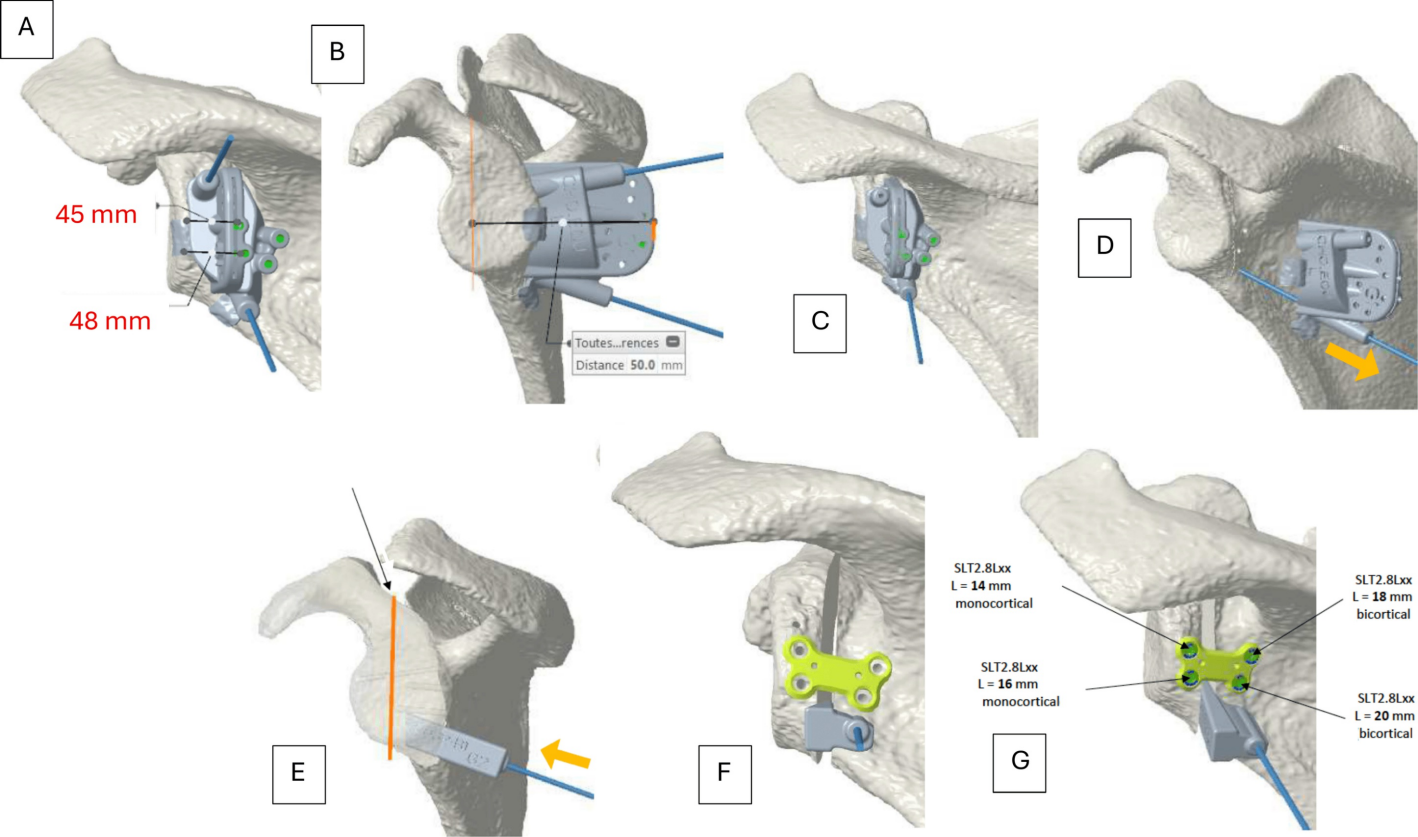
Step-by-step surgical technique (A) Positioning of the guide and the two pins in the posterior view and (B) in the lateral view. (C) Removal of the superior pin. (D) Withdrawal of the guide along the inferior pin, with the orange arrow indicating the direction of guide withdrawal. (E) Opening performed using a custom-made wedge, with the orange line indicating the cutting limit to preserve an adequate anterior hinge and the orange arrow indicating the direction of wedge insertion. (F) Placement of the plate. (G) Screw insertion after drilling.

Based on the 3D simulation, a custom cutting guide was designed to conform precisely to the posterior cortical surface of the glenoid and scapular neck. The guide incorporated dedicated contact points on non-eroded bone areas and integrated cylindrical sleeves to guide Kirschner wires for both temporary fixation and controlled osteotomy execution. The geometry of the guide ensured reproducible positioning, minimizing the risk of rotational or translational error. A virtual fit verification was performed on the 3D model before manufacturing. The guide was then sterilized according to manufacturer recommendations and prepared for intraoperative use (Figure 3).

Surgical technique

Patient Positioning

The patient is placed in the prone position, supported by thoracic and iliac pads to maintain stability. The upper limb is prepared and draped freely, allowing dynamic control of rotation and abduction throughout the procedure.

Surgical Approach

A posterior incision is centered along the posterior border of the acromion and extended caudally following the fibers of the posterior deltoid over approximately 8 cm. After incision of the skin and subcutaneous tissue, the posterior deltoid is partially detached from the posteroinferior edge of the scapular spine and retracted laterally. The long head of the triceps is identified medially, and the interval between the infraspinatus and teres minor muscles is exposed. The infraspinatus-teres minor interval is carefully developed, with attention to protecting the axillary nerve, located anterior and inferior to this plane. Meticulous hemostasis is achieved. Gentle retraction exposes the posterior capsule of the glenohumeral joint. A longitudinal or T-shaped capsulotomy is performed along the posterior rim of the glenoid, providing direct access to the posterior glenoid surface and adjacent scapular neck.

Anatomical Identification and Preparation

Under direct visualization, and, if necessary, assisted by fluoroscopic guidance, the posterior glenoid surface is exposed while preserving the posterior labrum, which may be tagged with a suture for protection. Adjacent soft tissues are cleared from the posterior cortical surface of the glenoid over a few millimeters to allow the accurate seating of the patient-specific cutting guide.

Placement of the Patient-Specific Cutting Guide

The custom-made guide is positioned on the posterior glenoid surface, matching the predefined anatomical landmarks (glenoid margins and scapular reliefs). Proper bone-to-guide congruence is confirmed visually and by palpation. Fixation pins are inserted through the designed holes to secure the guide. Positioning is verified against the preoperative plan (correction axis, depth, and orientation of the osteotomy).

Osteotomy Execution

The posterior osteotomy is performed along the trajectory defined by the guide using a fine oscillating saw. Gradual opening of the osteotomy is achieved with a dedicated distractor until the planned correction of glenoid retroversion is obtained.

Fixation and Final Assessment

**Figure 4:**
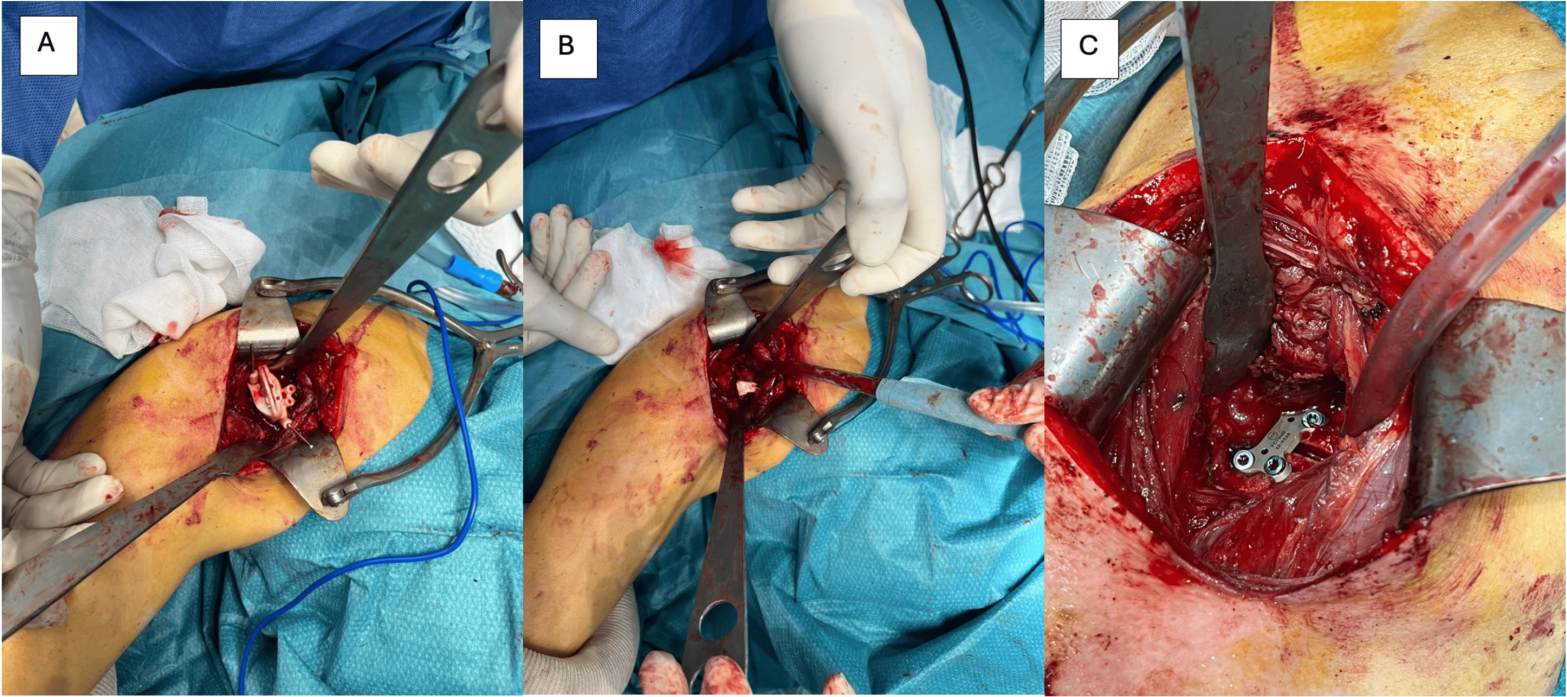
Intraoperative view of the posterior opening glenoid osteotomy (A) Placement of the cutting guide. (B) Opening performed using an osteotome. (C) Fixation with a four-hole 3.5 mm locking plate.

Fixation is achieved according to the preoperative plan, using a specific glenoid plate designed for the correction obtained. Intraoperative fluoroscopy is used to confirm the angular correction and optimal screw positioning. The posterior capsule is closed without tension. The posterior deltoid is reattached to the scapular spine, and the subcutaneous and skin layers are closed in standard fashion (Figure 4).

Postoperative Care

A compressive dressing and a shoulder sling are applied. Immobilization is maintained for four weeks, with assisted passive mobilization starting on the third postoperative day, avoiding internal rotation. Progressive active motion is initiated between the fourth and sixth weeks, according to radiographic consolidation and clinical tolerance.

## Discussion

The posterior opening glenoid osteotomy aims to correct excessive retroversion, which is recognized as a major factor in posterior humeral subluxation and posterior shoulder instability [3]. The technique described in this work builds upon the anatomical approaches initiated by Scott while integrating recent advances in 3D planning and patient-specific cutting guides (PSI).

Results and contribution of 3D planning

In our experience, 3D planning allows for the precise correction of glenoid version while optimizing the location of the osteotomy [12]. The ability to virtually simulate the correction and to print an adjusted cutting guide enhances the reproducibility of the procedure, reduces the risk of orientation errors, and increases safety when performing cuts in anatomically complex regions [13]. The use of PSI enables the faithful execution of the preoperative plan, thereby limiting inter-operator variability reported in series relying solely on visual or fluoroscopic guidance [14].

Comparison with the literature

The initial series by Scott and Graichen et al. reported satisfactory clinical outcomes [4,5], though at the cost of glenohumeral osteoarthritic progression in nearly one quarter of cases. Waltenspül et al. demonstrated, with more than 15 years of follow-up, that isolated correction of retroversion is insufficient to restore normal glenohumeral biomechanics, highlighting the importance of considering the scapula as a whole [6]. Integrating acromial parameters into 3D planning, as in our protocol, allows the assessment of whether an associated correction is required when the acromion is high and horizontal, a morphotype frequently observed in posterior instabilities. This tridimensional approach is consistent with the recent work of Gerber et al., who proposed a combined morphological correction of both the glenoid and the acromion [12].

Strengths and limitations of the technique

The main advantages of this technique lie in its geometric precision and the improved safety of the procedure afforded by the patient-specific guide. Targeted correction of retroversion can be achieved without excessive elongation of the scapular neck, and fixation with a low-profile locking plate allows for early mobilization. However, this approach remains technically demanding and requires an appropriate infrastructure-specific imaging protocol, dedicated planning software, and a guide manufacturing time of several weeks. The osteotomy corrects version but does not address pre-existing cartilage damage, which may explain the arthritic progression observed in long-term series. Moreover, the current limited clinical follow-up does not yet allow the assessment of the long-term maintenance of correction or its overall functional impact.

## Conclusions

The 3D-planned posterior opening glenoid osteotomy using a patient-specific cutting guide represents a logical and safe evolution of anatomical techniques for correcting glenoid retroversion. This approach enhances accuracy and reproducibility in the execution of the osteotomy. However, the data presented here relate exclusively to the technical feasibility and precision of surgical planning and not to long-term clinical outcomes. This technique should therefore be considered within the broader context of a comprehensive three-dimensional assessment of scapular morphology, including acromial architecture and humeral head centering. Longer-term clinical studies will be necessary to determine the durability of the correction and its functional implications.
